# SNP genotyping using TaqMan® technology: the CYP2D6*17 assay conundrum

**DOI:** 10.1038/srep09257

**Published:** 2015-03-19

**Authors:** Andrea Gaedigk, Natalie Freeman, Toinette Hartshorne, Amanda K. Riffel, David Irwin, Jeffrey R. Bishop, Mark A. Stein, Jeffrey H. Newcorn, Lazara Karelia Montané Jaime, Mariana Cherner, J. Steven Leeder

**Affiliations:** 1 Division of Clinical Pharmacology, Toxicology & Therapeutic Innovation, Children's Mercy Kansas City, Kansas City, MO; 2 Department of Pediatrics, University of Missouri-Kansas City, Kansas City, MO; 3 Tanenbaum Centre for Pharmacogenetics, Centre for Addiction and Mental Health, Toronto, ON, Canada; 4 Thermo Fisher Scientific, Genetic Analysis, South San Francisco, CA; 5 Department of Experimental and Clinical Pharmacology, University of Minnesota, Minneapolis, MN; 6 Department of Psychiatry and Behavioral Sciences, University of Washington, Seattle, WA; 7 Department of Psychiatry, Mount Sinai School of Medicine, New York, NY; 8 The University of The West Indies, Department of Paraclinical Sciences, St. Augustine, Trinidad and Tobago; 9 Department of Psychiatry, University of California San Diego, La Jolla, CA

## Abstract

CYP2D6 contributes to the metabolism of many clinically used drugs and is increasingly tested to individualize drug therapy. The *CYP2D6* gene is challenging to genotype due to the highly complex nature of its gene locus. TaqMan® technology is widely used in the clinical and research settings for genotype analysis due to assay reliability, low cost, and the availability of commercially available assays. The assay identifying 1023C>T (rs28371706) defining a reduced function (CYP2D6*17) and several nonfunctional alleles, produced a small number of unexpected diplotype calls in three independent sets of samples, *i.e.* calls suggested the presence of a CYP2D6*4 subvariant containing 1023C>T. Gene resequencing did not reveal any unknown SNPs in the primer or probe binding sites in any of the samples, but all affected samples featured a trio of SNPs on their CYP2D6*4 allele between one of the PCR primer and probe binding sites. While the phenomenon was ultimately overcome by an alternate assay utilizing a PCR primer excluding the SNP trio, the mechanism causing this phenomenon remains elusive. This rare and unexpected event underscores the importance of assay validation in samples representing a variety of genotypes, but also vigilance of assay performance in highly polymorphic genes such as *CYP2D6*.

Cytochrome P450 2D6 (CYP2D6) is one of the most scrutinized phase I drug metabolizing enzymes due to its involvement in the metabolism and bioactivation of 20 to 25% of clinically used drugs. Among the long list of drugs and substrates are many antidepressants and antipsychotics, pain medications such as codeine and tramadol, the estrogen receptor antagonist tamoxifen, and also drugs of abuse^1^,^2^,^3^. CYP2D6 activity varies widely in most populations with the potential to affect the ability of individuals to metabolize or bioactivate medications. To date, over 100 allelic variants and subvariants have been described that give rise to poor (PM), intermediate (IM), extensive (EM) and ultrarapid (UM) metabolizer phenotypes^4^,^5^. Dose-related adverse events are most prominent in individuals lacking CYP2D6 activity (PMs) or having extremely fast (UM) metabolism. Hence, *CYP2D6* genotype testing is increasingly utilized to predict a subject's metabolizer status to individualize drug choice and dosing, especially in psychiatry^6^,^7^ and pain management using opioids^8^,^9^,^10^. Furthermore, the importance of *CYP2D6* pharmacogenetics is also highlighted by Clinical Pharmacogenetics Implementation Consortium (CPIC) guidelines for tricyclic antidepressants^11^ and codeine^12^.

Due to the complexity of the human *CYP2D* locus and the highly polymorphic nature of the *CYP2D6* gene itself, *CYP2D6* genotype analysis not a trivial undertaking. Two recent reviews highlight the complexity of *CYP2D6* gene analysis, interpretation of results, and the challenges of predicting phenotype from genotype data^13^,^14^. A fundamental issue for any genetic test, whether it is performed in the research or clinical setting, is the accuracy of the reported test results, *i.e.* what sequence variations are present and which are not, as all subsequent interpretations (genotype call, prediction of metabolizer status, drug dose recommendation) are based on the test result.

At present, there are numerous commercial platforms, methods, and assays available for *CYP2D6* pharmacogenetic (PGx) testing^6^,^13^,^15^,^16^ including drug metabolism genotyping analysis using TaqMan assays^17^. TaqMan Drug Metabolism Genotyping Assays use 5′ nuclease assay chemistry to detect specific SNP, multinucleotide polymorphism, and insertion/deletion alleles. A relatively small region flanking the target SNV is amplified using locus-specific primers and alleles are detected using two TaqMan® MGB probes labeled with VIC® dye or FAM™ dye. Designing TaqMan assays to *CYP2D6* gene variants is challenging due to its highly polymorphic nature and homology to pseudogenes. Assays are designed using an algorithm pipeline that includes masking of non-target SNPs in input SNV context sequences and in silico QC of assay designs to avoid underlying polymorphisms and to ensure high target specificity^18^, as assays having primers and/or probes that anneal to sequences containing polymorphic sites or pseudogenes may lead to erroneous calls in samples carrying these SNVs^19^.

Several DNA samples with an unusual and unexpected diplotype were independently observed at three academic centers in the US and Canada. These samples all presented with heterozygous calls for the CYP2D6*4-defining SNP 1846G>A and homozygous calls for the CYP2D6*17-defining SNP rs28371706 (1023C>T) when genotyped with TaqMan assays. Allele nomenclature in this report is according to the Human Cytochrome P450 (CYP) Allele Nomenclature Database^20^. 1023C>T is the signature SNP for CYP2D6*17, a reduced function allele which is commonly detected in subjects of sub-Saharan origin. This SNP, however, is also part of the haplotype of the non-functional allelic variants CP2D6*40 and *58 as well as CYP2D6*64 and *82, which have been tentatively assigned reduced or non-functional status. For simplicity, we will refer to the TaqMan assay detecting the 1023C>T SNP as the ‘CYP2D6*17 TaqMan assay’.

To date, however, 1023C>T has not been reported as a subvariant of CYP2D6*4 as no allele(s) have been described that contain both 1023C>T and 1846G>A as implied by the unexpected TaqMan assay results. Subsequent gene resequencing did not reveal the presence of any known or novel SNVs in the TaqMan primer or probe regions. Observation of this phenomenon by several independent groups in samples from multiple studies implied that the underlying genetic context may be relatively common, and warranted further investigation.

As a common cause of unexpected homozygosity is mono-allelic amplification, the goal of this study was to characterize the apparent ‘drop out’ of the CYP2D6*4 allele in the 1023C>T CYP2D6*17 TaqMan assay. Understanding the underlying causes leading to this phenomenon is crucial in solving the conundrum at hand, but are also invaluable for explaining similar unpublished observations by other investigators for this or other TaqMan assays and for primer-based assay designs at large, and ultimately for accurate phenotype assignment in a clinical setting.

## Results

The cases described in this report were genotyped for a number of sequence variations with TaqMan assays to determine *CYP2D6* genotype. Nineteen of the cases tested homozygous for the 1023C>T SNP, which was inconsistent with known haplotypes. DNA quality and/or concentration issues were ruled out as the samples in question amplified equally well compared to all other samples within a run. Contamination issues were initially considered and DNA re-isolated from the first cases observed at CMH; this, however, did not resolve the issue. As additional cases with the same miscall pattern were identified and this phenomenon was also observed in samples genotyped by independent laboratories, contamination was no longer deemed to be a likely explanation for the inconsistent genotype calls.

**Figure 1:**
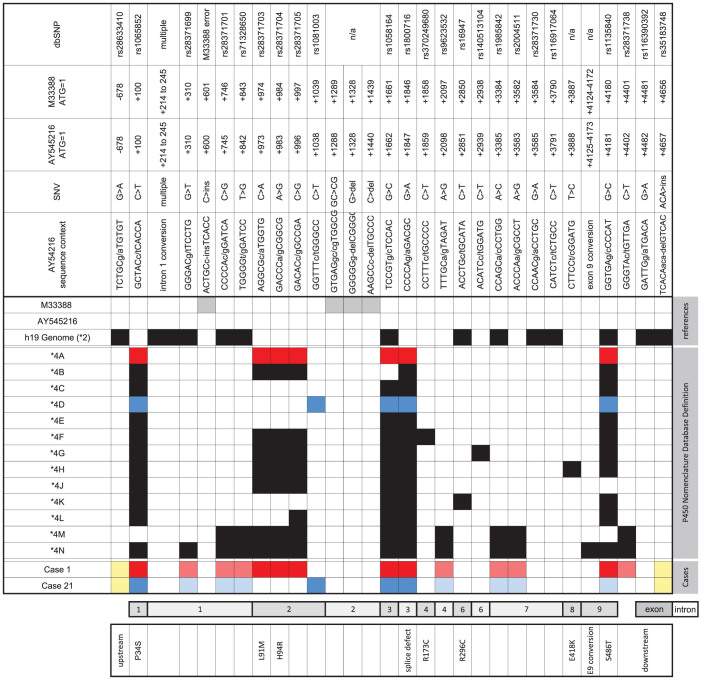
CYP2D6*4 subvariants. The top panel provides details about the SNVs including rs numbers, positions relative to the numbering system on which the allele nomenclature is based (M33388) and the widely accepted CYP2D6*1 reference sequence AY545216, the nature of the SNV, and sequence context. The presence of the intron 1 conversion event is characterized by multiple SNPs (rs1080995, rs267608284, rs108996, rs74644586, rs75276289, rs28695233, rs149744965, rs56011157). The middle ‘checkerboard’ panel shows SNVs relative to the AY545216 reference. Variation is highlighted by colored boxes. Gray boxes denote sequence errors in M33388. For general information, sequence differences to Genome Build GRCh37 that harbors a CYP2D6*2, is shown. Lines *4A through *4N depict a graphical representation of the P450 Nomenclature Database^20^ for these alleles. Note that *4A-K lacks any annotations for intronic regions. Sequence variations found in cases 1 and 21 that match with CYP2D6*4A and *4D are highlighted in red and blue, respectively. Intronic SNPs in the cases are shown in light red and blue as these are not represented in the nomenclature-defined variants. Cases were sequenced between the SNPs shown in yellow. The bottom panels provide location of SNV regarding to exon and intron and consequence of SNV.

Twenty-five subjects including the 19 cases with inconsistent results for the 1023C>T SNP were selected for further investigation and were grouped as following: Each individual possessed a CYP2D6*17 allele (defined by the presence of the SNP at position 1023) and a CYP2D6*4 allele (defined by the presence of SNPs at positions 100 and 1846). Cases 1–18 presented with inconsistent (i.e. homozygous) calls for 1023C>T; these subjects were eventually found to carry a CYP2D6*4 subvariant with the SNP trio. Cases 19 and 20 had consistent calls for 1023C>T; both subjects revealed the CYP2D6*4D subvariant which does not possess the SNP trio. Cases 21–25 carried a CYP2D6*4 gene duplication; four were typed heterozygous for 1023C>T similar to cases 19 and 20, while one was homozygous. An overview of CYP2D6*4 haplotypes (subvariants) is provided in Figure 1. The following paragraphs provide additional details about the findings for each group.

### Cases 1–18

**Figure 2:**
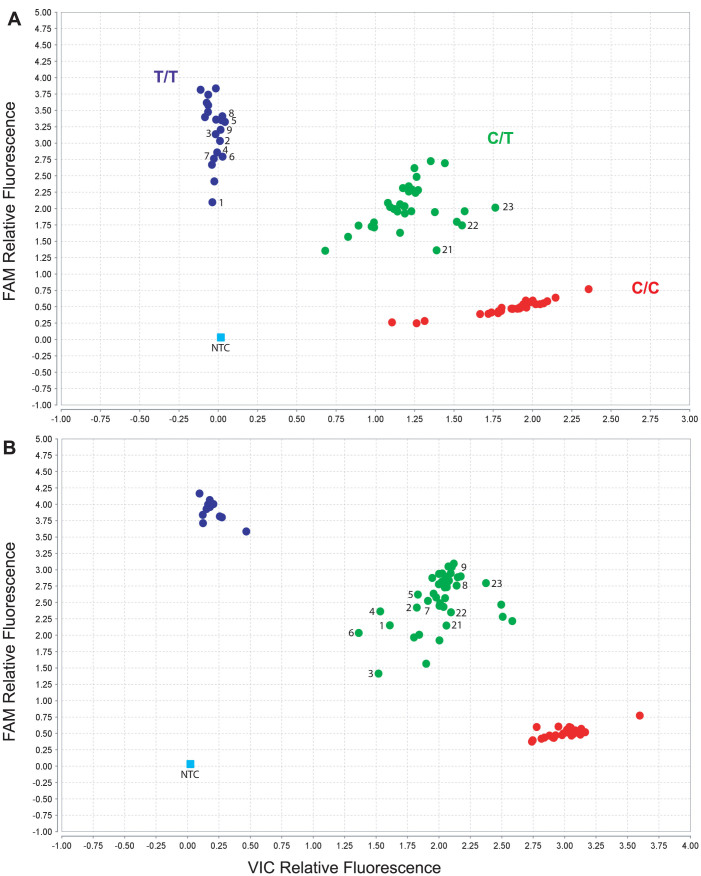
CYP2D6*17 (1023C>T) TaqMan Assay Genotype Results. (Panels A and B) show the scatter plots for TaqMan assays C__2222771_40 (original assay) and C__2222771_A0 (alternative assay). Numbered subjects correspond to cases 1–9 and 21–23 (Tables 1 and 2). Cases 1–9 were identified as 1023 T/T with the original assay (panel A), but genotyped accurately as 1023 C/T with the alternative assay (panel B). Additional samples of known genotypes were analyzed as references to allow cluster formation in the scatter plots; none of these controls carry a CYP2D6*4.

*CYP2D6* genotyping performed at three different institutions identified 16 subjects of predominantly African ancestry (Table 1) which presented heterozygous for certain key SNPs (100C>T, 1846 G>A, 2850C>T), were negative for all other SNPs tested as well as copy number variations (CNVs), but were homozygous for 1023T/T (Figure 2A). Cases 1–11 also did not exhibit any evidence for the presence of hybrid genes (*CYP2D6/2D7* or *2D7/2D6*) based on quantitative CNV analysis that could conceivably interfere with the assay result in question. This suggested that these subjects have a CYP2D6*4/*17 genotype with a novel CYP2D6*4 subvariant that carries the 1023C>T SNP. When using a CMH custom-made CYP2D6*17 TaqMan assay these subjects also genotyped homozygous for 1023T/T (data comparable to those shown in Figure 2A). Two Coriell samples (cases 12 and 13) with the same SNP patterns were identified by Thermo Fisher Scientific (formerly Life Technologies, Foster City, CA, USA). Seven subjects with homozygous 1023T/T calls by TaqMan assay were also interrogated by RFLP analysis. In contrast to the TaqMan assay results, RFLP indicated heterozygosity for the CYP2D6*17 1023C>T SNP (Table 2).

This result was confirmed by complete (case 1) or partial (cases 2–11) gene resequencing. Sequencing was performed on PCR products representing both alleles; no novel SNPs that could conceivably interfere with the TaqMan assay were found. It was noted however, that all subjects had three SNPs (974C>A, 984A>G and 997C>G) that are commonly found on CYP2D6*4 variants including *4A, B, F, H, J, and *M* while *4L is defined as having 997C>G only and *4C, D, E, and *K* appear to lack this SNP trio (Fig. 1, Table 1 and Ref. 20).

### Cases 19 and 20

Cases 19 and 20 tested heterozygous for 100C>T, 1023C>T, 1846G>A and 2850C>T by TaqMan, which is consistent with a CYP2D6*4/*17 genotype. In contrast to cases 1–18, there were no conflicting genotyping results (Figure 2A and Table 2) suggesting that these two subjects did not have any SNV(s) affecting assay performance. Interestingly, sequencing revealed that case 19 was wild-type for 974C>A, 984A>G and 997C>G providing evidence that the SNP trio is suppressing amplification from the CYP2D6*4 subvariants carrying these three SNPs. Sequence information was not available for case 20. Both cases were also negative for CNVs and were negative for hybrid arrangements.

### Cases 21–25

These five cases were positive for a gene duplication event. A duplication on the CYP2D6*4 allele (CYP2D6*4×2) was defined in cases 21–23 by genotyping a fragment that was specifically generated from the duplicated gene (fragment D). The duplication was not further specified in cases 24 and 25. As shown in Table 2 and Figure 2A, four of these cases were genotyped heterozygous for 1023C>T and 1846G>A by TaqMan while case 25 was homozygous 1023T/T and heterozygous 1846G/C. The CMH custom-made TaqMan assay also showed heterozygosity for 1023C>T for cases 21–23.

### Characterization of the CYP2D6*4 allele

The preliminary sequencing results described above for cases 1–11 and 19 suggested that the presence of 974C>A, 984A>G and/or 997C>G interfered with the CYP2D6*17 TaqMan assay by preventing amplification and accurate detection of 1023C (wild-type) on CYP2D6*4 when this allele was paired with CYP2D6*17. As a result, fluorescence was only produced from the 1023T-containing CYP2D6*17 allele mimicking a homozygous signal. To unequivocally determine the CYP2D6*4 haplotype for the exon 2 region in which these SNPs reside, allele-specific long-range PCR and sequencing was performed on CMH cases 1–9. Cases 1–3 and 5–9 indeed carried all three SNPs on their CYP2D6*4 allele; sequence analysis for cases 10–13 and 19 was performed on diploid template and identified the presence of the three SNPs and were tentatively assigned to the CYP2D6*4 allele. In contrast, these SNPs were absent in CMH cases 21–23. However, another SNP, 1039C>T, was detected in the latter cases by gene resequencing which allowed us to subtype their CYP2D6*4 allele as CYP2D6*4D. Subjects for which no information was available for 1039C>T, but genotyped correctly for 1023C>T (cases 20 and 24) were tentatively assigned as having a CYP2D6*4D allele.

The CYP2D6*4 alleles of cases 1 and 21 were completely sequenced. As shown in Figure 1, these matched with allele definitions CYP2D6*4A and *4D, respectively, not considering intronic SNPs. To the best of the authors' knowledge, the majority of CYP2D6*4 subvariant definitions are based on exon and exon/intron junction sequences only; hence many do not have any annotations for SNVs located in introns or the 3′-and 5′-flanking regions.

Partial resequencing of the duplicated CYP2D6*4 gene of cases 21–23 also determined absence of the 974C>A, 984A>G and 997C>G SNP; cases 24 and 25 were not available for sequence analysis.

### Review of the commercial CYP2D6*17 TaqMan assay and alternative assay design

**Figure 3:**
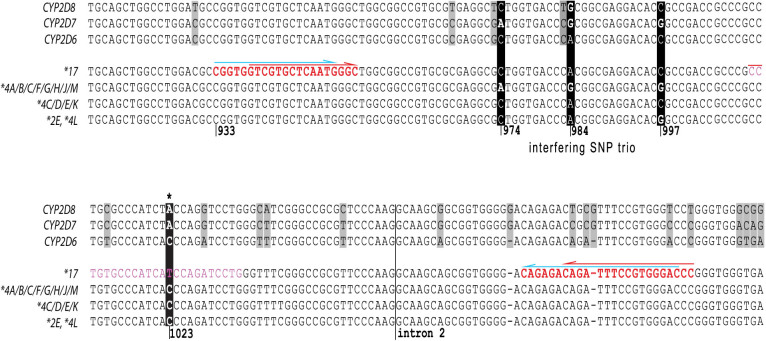
Sequence Comparison of *CYP2D6* variants, *CYP2D7* and *CYP2D8*. Each line represents the nucleotide sequence for a given gene or genetic variant. The key CYP2D6*17 SNP is highlighted by a black background and designated by an asterisk *. The tentatively interfering SNP trio is also indicated in the top panel by a black background; differing positions are highlighted (CYP2D6*4A, B, C, F, G, H, J and *M* have the SNP trio, while *4C, D, E and *K* do not) *2E and *4L contain only the SNP at position 997. Light grey boxes emphasize differences between *CYP2D6, 2D7* and *2D8*. Regions targeted for PCR primer and probe binding are shown in bold red type and plain green type, respectively. The red arrows above red letters denote the primer binding sites for the original (C__2222771_40) TaqMan assay while blue arrows indicate the primer sequences utilized for the CMH custom-made TaqMan assays. The probe binding site for the latter is shown by green letters.

In order to resolve the CYP2D6*17 conundrum, we engaged in a collaboration with Thermo Fisher. The PCR primers of the CYP2D6*17 assay ID C___2222771_40 are located upstream of the 974C>A, 984A>G and 997C>G SNP trio and the fluorescent probes bind downstream of 997C>G, which is similar in design compared to the custom-made assay developed at CMH (Figure 3). Thermo Fisher provided for testing an alternative CYP2D6*17 assay design (C___222771_A0), which differed from C___2222771_40 in the position of just the upstream primer, which was located between the 997C>G and target 1023C>T SNP. It had been observed at Thermo Fisher that the alternate assay genotyped two Coriell samples (NA17116 and NA17121, cases 12 and 13) as heterozygous for the CYP2D6*17 SNP (1023C/T) whereas the original assay genotyped these as homozygous for CYP2D6*17 (1023T/T); the two samples were also genotyped as heterozygous for CYP2D6*4. This phenomenon was not understood as there were no known SNPs within the primer or probe binding sites and sequence analysis had not revealed novel underlying SNPs. Similar to the list of mis-genotyped samples accumulated at the three study sites, sequencing revealed that these samples were indeed heterozygous for CYP2D6*17 and carried the 974C>A, 984A>G and 997C>G SNP trio. As shown in Figure 2B and summarized in Table 2, all samples which initially genotyped as 1023T/T, were correctly identified as 1023C/T with the alternative assay (C___2222771_A0). Samples were confirmed independently at all three study sites.

**Figure 4:**
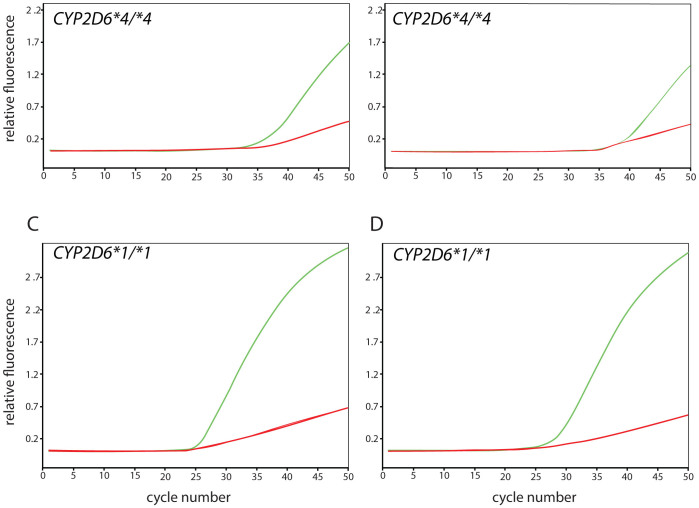
Real-time amplification for the original and alternate CYP2D6*17 TaqMan assays. Fluorescent signals detected for individuals with CYP2D6*4/*4 and CYP2D6*1/*1 genotypes. (Panels A and C) represents the original TaqMan assay (C__222771_40), (panels B and D) the alternate assay C__2222771_A0). The green curves indicate amplification from the ‘C’ allele (wild-type/reference, 1023C or CYP2D6*17) while the green curve represents the ‘T’ allele (variant, 1023 T, CYP2D6*17 or other variant haplotype containing this SNP).

As shown in Figure 4, samples with a CYP2D6*4/*4 genotype (SNP-trio positive) did amplify with the original assay and were accurately called by the TaqMan® Genotyper™ Software. Upon manual interrogation of amplification plots, it appears that there is a trend towards lower efficiency of amplification when comparing CYP2D6*4/*4 with CYP2D6*1/*1 samples. The trend towards lower amplification, is, however, still within the variability seen within a typical sample panel. Therefore, the drop-out phenomenon did not interfere with amplification to an extent that would prevent allele calling.

An analysis of the secondary structure of the TaqMan assay PCR products using mfold determined seven possible structures with melting temperatures ranging from 64.1°C to 67.7°C for a CYP2D6*17-derived PCR product which was lower compared to those calculated for CYP2D6*4 with the SNP trio (three structures, ranging from 65°C to 69.3°C) and CYP2D6*4D (five structures) ranging from 68.5°C to 70.4°C.

## Discussion

A growing number of subjects were observed over time which consistently presented homozygous for 1023C>T (CYP2D6*17) and heterozygous for 1846G>A (CYP2D6*4) by TaqMan genotyping. Since this SNP pattern was inconsistent with the definition of known allelic variants, *i.e.* an allele that carries 1023T and 1846A, a genotype assignment could not be made with confidence. We initially suspected that a novel SNP interfered with the TaqMan assay and prevented the generation of a fluorescent signal from the second allele, specifically when the second allele was a CYP2D6*4. CYP2D6*4 drop-out would trigger the CYP2D6*17-derived signal to appear homozygous. A drop-out event was further supported by hetero-zygous results for 1023C>T by RFLP analysis using an assay that employs PCR primers located more distant from the SNP and having different binding sites than the commercial and CMH-designed TaqMan assays, respectively. However, the presence of a novel sequence variation could not be substantiated by sequence analysis of the gene region of interest, although we confirmed heterozygosity for 1023C>T and found a trio of three SNPs that is commonly found in CYP2D6*4 haplotypes^20^ (Figures 1 and 3). In addition, we were unable to resolve the issue with a custom TaqMan assay we designed. Eventually, we encountered additional samples (cases 19–24) that produced the expected pattern of heterozygosity for 1023C>T in the presence of CYP2D6*4. The four cases available for sequencing all lacked the SNP trio upstream of 1023C>T turning our attention to these SNPs as potential culprits.

To solve the assay issue, a collaboration with Thermo Fisher was established. The commercially available and CMH custom-designed CYP2D6*17 TaqMan assays employed similar, overlapping PCR primer binding sites, explaining their comparable performance. These primer binding sites were chosen to ensure that PCR product is only generated from *CYP2D6* and discriminates against *CYP2D7* and *CYP2D8* (Figure 3), avoid known SNPs being located within primer binding sites as well as keep the PCR fragment within length constraints for assay efficiency. To demonstrate that the SNP trio interfered with assay performance, possibly by considerable less efficient amplification of the CYP2D6*4 allele leading to CYP2D6*4 allele drop-out, an alternative assay excluding the SNP trio from the amplification product was provided by Thermo Fisher for further testing. The redesigned assay correctly genotyped all subjects for 1023C>T (Table 2 and Figure 2B) implicating the SNP trio as the underlying cause for the miscalls.

The mechanism by which the SNP trio found on the CYP2D6*4 allele interferes with CYP2D6*17 assay amplicon generation and causes CYP2D6*4 drop-out (*i.e.* CYP2D6*4 alleles are not amplified) remains elusive. The first of these SNPs (974C>A) is about 20 bp downstream of the 3′ end of the forward PCR primers of the commercial and custom-made assays and should therefore not impact amplification efficiency. Also, subjects homozygous for CYP2D6*4 (SNP trio positive) are relatively common (*e.g.* Coriell DNAs NA17123, NA17225, NA17226) and do amplify and genotype correctly with the original assay (Figure 4). This suggests that the amplification of the CYP2D6*4 chromosome is only impacted when it carries the SNP trio and when paired with a chromosome that lacks the trio. Furthermore, the drop-out event is only recognized in subjects heterozygous for CYP2D6*4 (SNP trio-positive) and CYP2D6*17. CYP2D6*4 allele drop-out would not be apparent in subjects with, for example, CYP2D6*1/*4, *2/*4 or *4/*10 and other *4/non-*17 genotypes, because CYP2D6*4 and non-*17 alleles are all wild-type for 1023C>T and thus genotype “accurately”.

Based on the frequencies of the CYP2D6*4 and *17 allelic variants in African and African American populations^12^ of 3–6% and about 18%, respectively, approximately 1–2% of subjects in these populations are expected to have a CYP2D6*4/*17 genotype, which is consistent with the number of subjects we have observed in our study populations. This phenomenon was likely observed by others as well, for instance Friedrich et al.^21^ list a number of alleles that were not further characterized and reported as ‘others’ while commercial service laboratories may simply report such cases as ‘no-calls’ or ‘undetermined’. Although we were not able to fully explain the mechanistic causes of the drop out phenomenon, our findings will allow other investigators to go back and resolve some of their cases, and also allow researchers as well as commercial laboratories to accurately detect CYP2D6*4/*17 genotypes with the redesigned assay moving forward.

The occurrence of the allele amplification drop-out we describe in this report was unexpected. Interactions between SNP(s) that are not within or next to primer binding sites are generally not considered to be of concern when designing TaqMan or other PCR-based genotyping assays. Often, especially when working with highly polymorphic genes such as the cytochrome P450s, choices for primer and/or probe locations are rather limited and restricted to certain areas as presented in Figure 3 for *CYP2D6, 2D7* and *2D8*. TaqMan Drug Metabolism Genotyping Assays undergo stringent bioinformatic and wet lab testing quality control before being commercialized by Thermo Fisher. As well, concordance experiments with samples geno-typed by other technologies have demonstrated their overall high accuracy and reproducibility^18^. Moreover, we have successfully geno-typed a large number of individuals for a series of CYP2D6 polymorphisms and not encountered any problems regarding assay accuracy, reproducibility or specificity. Thus, amplification interference by SNPs that do not underlie primer or probe target sites appears to be a rare event. Allele drop-out due to a close-by SNP or SNP combination may not be limited to TaqMan technology, but could conceivably occur in any assay amplifying relatively short PCR products. It is therefore of utmost importance to thoroughly test assay performance on a wide range of genotypes that are ideally sequence-confirmed as well as be vigilant when reviewing assay results, as we have demonstrated in this report.

The determination of the mechanism behind the allele amplification drop-out could enable avoiding primer design to regions that may be susceptible to this phenomenon. We speculated that Taq polymerase properties may contribute to the preferred amplification of one allele in the presence of the SNP trio that is found on the majority of CYP2D6*4 alleles. To that end, we tested amplification reagents from other suppliers; however, none was able to overcome the allele-selective amplification problem. The SNP trio could conceivably also facilitate secondary structures which melt at a higher temperature and are therefore amplifying less efficiently compared to alleles lacking these SNPs. Analysis with mfold determined that the PCR product generated from the CYP2D6*17 allele have lower Tm values for tentative secondary structures compared to CYP2D6*4 with the SNP trio. However, secondary structures of the PCR fragment from CYP2D6*4D (without the trio) had the highest Tm values. Should secondary structures indeed be causative further modeling and experimentation is needed to define the mechanism of the CYP2D6*4 subtype allele drop-out phenomenon.

## Methods

All methods and procedures were carried out in accordance with approved guidelines.

### Subjects

All DNA samples for this analysis were obtained from individuals participating in IRB-approved studies and provided written informed assent or consent. Study protocols under which *CYP2D6* genotyping was performed were approved by the Institutional Review Boards of the Children's Mercy Hospital (CMH), Kansas City, MO, USA (n = 2), Mount Sinai School of Medicine, NY, NY, USA and the University of Illinois at Chicago (UIC), Chicago, IL, USA (n = 8), the University of California, San Diego, CA, USA (n = 2), the Ethics Committee of the Faculty of Medical Sciences, University of the West Indies, Trinidad and Tobago (n = 5), and the Research Ethics Board at the Centre for Addiction and Mental Health (CAMH), Toronto, ON, Canada (n = 3). Two liver tissue samples were from the National Institute of Child Health and Human Development-supported tissue retrieval programs at the Central Laboratory for Human Embryology at the University of Washington (Seattle, WA). The use of these tissues was classified as non-human subject research by the University of Missouri-Kansas City Pediatric Health Sciences Review Board (MO, USA). In addition, three DNA samples from the Coriell Institute for Medical Research (Camden, NJ) were investigated. Table 1 summarizes additional information including sample test site (institution where genotyping was performed), source of genomic DNA, and ethnicity.

Twenty-two additional previously genotyped subjects (16 negative for CYP2D6*17, 4 heterozygous and 2 homozygous, respectively) served as control samples to evaluate the performance of TaqMan assays.

### *CYP2D6* genotyping with TaqMan

High quality genomic DNA (gDNA) was isolated from whole blood and tissues using silica-based spin columns such as the QIAamp® DNA Blood Mini Kit, DNeasy® Blood and Tissue Kit from Qiagen (Valencia, CA) or by a phenol-chloroform-based procedure^22^. Saliva samples were collected from participants at CAMH in Oragene DNA kits (DNA Genotek, Kanata, ON, Canada). Total gDNA was extracted from the preserved saliva using the chemagen MSM I automated DNA extractor (Perkin-Elmer, Waltham, MA) 4 mL saliva option as per supplier's instructions. For UIC study participants, genomic DNA was extracted from whole blood samples using a PureGene® kit purchased from Qiagen (Valencia, CA).

*CYP2D6* genotype analyses were carried out with ready-to-order TaqMan assays from Thermo Fisher (Table 3). For the purpose of this report, details are only provided for the assays testing the key SNPs utilized to identify CYP2D6*2 (rs16947, 2850C>T, assay ID C__27102425_10), CYP2D6*4 (rs3892097, 1846G>A, assay ID C__27102431_D0), CYP2D6*4 and *10 (rs1065852, 100C>T, C__11484460_40) and CYP2D6*17 (rs28371706, 1023C>T, assay ID C___2222771_40; alternative assay ID C___2222771_A0), although all subjects were tested for additional allelic variants. Allele designation is according to the Human Cytochrome P450 (*CYP*) Allele Nomenclature Database^20^.

At CAMH, 20 ng gDNA was amplified as per manufacturer's directions scaled to a total volume of 10 μL in an Applied Biosystems® Veriti® 384-well thermal cycler for each of the above assay IDs. Post-amplification products were analyzed on an Applied Biosystems® ViiA™ 7 Real-Time PCR System and genotype calls were determined manually by comparison to six No Template Controls.

At UIC 15 ng gDNA was amplified as per manufacturer's directions scaled to a total volume of 10 μL in 96 well plate format using an Applied Biosystems® StepOnePlus™ Real-Time PCR System for each of the above assay IDs. Genotype calls were assessed with Applied Biosystems® TaqMan® Genotyper™ Software.

At CMH, study subjects were initially genotyped by pre-amplifying the *CYP2D6* gene by XL-PCR (referred to as fragment A, Table 3)^23^. The fragment was diluted 1000–2000-fold and 0.8 μl served as template for TaqMan genotyping reactions. Eight μl reactions were carried out in 96-well plates using the TaqMan® Genotyping Master Mix as recommended by Thermo Fisher. Cycling was performed on the Applied Biosystems® 7900 Real Time PCR System according to manufacturer's specifications and data analyzed with the SDS2.4 software.

All samples with discordant results with the original TaqMan assay (CYP2D6*17, rs28371706, 1023C>T, assay ID C___2222771_40) were repeated at least once.

A custom TaqMan assay for *CYP2D6* 1023C>T was developed at CMH. Primer and probe locations are shown in Figure 3. Assay conditions were optimized on samples with sequenced-confirmed genotypes.

### *CYP2D6* genotyping with RFLP

Selected study subjects were re-genotyped at CMH employing a restriction fragment length polymorphism (RFLP) assay. gDNA or diluted XL-PCR fragment A served as template to amplify a 414 bp long product with primers 5′ CTCCATCACAGAAGGTGTGACC (forward) and 5′ GCTCGGACTACGGTCATCAC (reverse). The amplicon was subsequently subjected to restriction digestion with *Fok*I for one hour and restriction fragments resolved by agarose gel electrophoresis. Amplicons derived from CYP2D6*17 (1023 T) remained uncut while those carrying wild-type 1023 C were cut once resulting in 392 bp and 23 bp long digestion products.

### *CYP2D6* gene re-sequencing

To generate the XL-PCR sequencing templates, KAPA Long Range Hotstart Ready Mix with dye containing dNTPs and polymerase (KAPA Biosystems, Woburn, MA, USA) was used at a 1× concentration and combined with 10–20 ng of genomic DNA, 0.5 μM of each primer, and 5% DMSO. Reactions were cycled for an initial denaturation at 94°C for 3 minutes followed by 35 cycles of denaturation (94°C for 20 sec), annealing (30 sec) and extension (details see Table 3). Subsequently, XL-PCR products were purified using a GenElute PCR Clean-Up Kit (Sigma, St. Louis, MO, USA). XL-PCR fragment A (generated from both chromosomes), fragment D (representing the duplicated gene only) and a CYP2D6*4-specific XL-PCR fragment that was generated with a CYP2D6*4-specific forward primer, were sequenced in both directions with BigDye® terminator v3.1 cycle sequencing chemistry and a 3730 capillary DNA analyzer (Thermo Fisher). GenBank entries AY545216 and M33388 served as reference sequences. Sequence primers utilized at CMH and covering the immediate gene regions of interest, *i.e.* 1023C>T and 1846G>A, are provided in Table 3. Coriell gDNA samples NA17116 and NA17121 were sequenced at Thermo Fisher on the Ion PGM™ System using a custom Ion AmpliSeq™ panel that contained *CYP2D6*-specific primers. CAMH samples were re-sequenced for the area surrounding 1023C>T as follows: 24 ng gDNA was combined with 1× Fermentas PCR buffer containing KCl (Fisher Scientific, Ottawa, ON, Canada), 1.5 mM Fermentas MgCl_2_, 0.2145 μg each primer (CYP2D6-17F and R, Table 2), 0.2 mM each dNTP (Fermentas), and 2 U Fermentas Taq polymerase in a total reaction volume of 50 μL. The PCR reactions were subjected to an initial denaturation for 5 min at 95°C, followed by 30 cycles of amplification in an AB 2720 thermal cycler: denaturing for 30 sec at 95°C, annealing for 30 sec at 60°C and extension for 30 sec at 72°C, and a final extension at 72°C for 10 min. Five μl of the PCR product was treated with 10 U Exonuclease I and 1 U FastAP Alkaline Phosphatase (Fisher Scientific, Ottawa, ON, Canada), incubated at 37°C for 15 min, followed by enzyme deactivation at 85°C for 15 min in an AB 2720. One μl of the sequencing template was amplified using the BigDye® terminator v3.1 cycle sequencing chemistry (Thermo Fisher) as per manufacturer's specifications. Sequencing products were purified using Centri-Sep spin columns (Princeton Separations, Adelphia, NJ, USA) following the manufacturer's directions and electrophoresed on a 3130 capillary DNA analyzer (Thermo Fisher) as per manufacturer's specifications. Sequencing products were aligned to a CYP2D6*1 reference sequence using SeqScape v2.5. Primer sequences are provided in Table 3.

### Secondary structure analysis

A 164 bp long DNA sequence framed by the primer sequences shown in red in Figure 3 was analyzed using the mfold Web Server at http://mfold.rna.albany.edu/. Mfold is a tool developed for predicting the secondary structure of RNA and DNA using thermodynamic methods^24^.

## Author Contributions

A.G. and N.F. conceived the project. A.G., N.F., T.H. and J.R.B. directed experiments at respective study sites. A.G., N.F., T.H., D.I., A.K.R. and J.S.L. analyzed and interpreted results. A.G., N.F., T.H. and A.K.R. prepared tables and figures. M.C., L.K.M.J., M.A.S. and J.R.N. provided sample materials and information. The manuscript was prepared by A.G. with critical contributions by T.H. and N.F. All authors reviewed, commented on and approved the final manuscript.

## Figures and Tables

**Table 1:** 

Case IDs	Genotyping performed at	DNA Source	Ethnicity
Case 1	CMH	blood	African American
Case 2	CMH	blood	African American
Case 3	CMH	blood	Afro-Trinidadian
Case 4	CMH	tissue	African American
Case 5	CMH	Coriell	African American
Case 6	CMH	blood	African American
Case 7	CMH	blood	African American
Case 8	CMH	blood	Afro-Trinidadian
Case 9	CMH	blood	Mixed/unknown
Case 10	CAMH	saliva	African American
Case 11	CAMH	saliva	Middle Eastern (Iraqi)
Case 12	Thermo Fisher	Coriell	African American
Case 13	Thermo Fisher	Coriell	African American
Case 14	UIC	blood	Hispanic
Case 15	UIC	blood	African American
Case 16	UIC	blood	African American
Case 17	UIC	blood	Biracial/Other
Case 18	UIC	blood	African American
Case 19	CAMH	saliva	Caucasian (Italian/German)
Case 20	UIC	blood	Caucasian
Case 21	CMH	blood	Afro-Trinidadian
Case 22	CMH	tissue	African American
Case 23	CMH	blood	Afro-Trinidadian
Case 24	UIC	blood	Biracial/Other
Case 25	UIC	blood	Biracial/Other

**Table 2:** Genotype and Gene Resequencing Results

	Genotype Data						Resequencing					
	TaqMan					RFLP	*4 haplotype					
Case ID	*2 2850 C>T	*4 1846 G>A	*10 100 C>T	*17 1023 C>T original	*17 1023 C>T alternate	*17 1023 C>T	974 C>A	984 A>G		997 C>G	1023 C>T	Genotype
Case 1	C/T	G/A	C/T	T/T	C/T	C/T	A	G		G	T	*4/*17
Case 2	C/T	G/A	C/T	T/T	C/T	C/T	A	G		G	T	*4/*17
Case 3	C/T	G/A	C/T	T/T	C/T	C/T	A	G		G	T	*4/*17
Case 4	C/T	G/A	C/T	T/T	C/T	C/T	A	G		G	T	*4/*17
Case 5	C/T	G/A	C/T	T/T	C/T	C/T	A	G		G	T	*4/*17
Case 6	C/T	G/A	C/T	T/T	C/T	ND	A	G		G	T	*4/*17
Case 7	C/T	G/A	C/T	T/T	C/T	ND	A	G		G	T	*4/*17
Case 8	C/T	G/A	C/T	T/T	C/T	C/T	A	G		G	T	*4/*17
Case 9	C/T	G/A	C/T	T/T	C/T	C/T	A	G		G	T	*4/*17
Case 10	C/T	G/A	C/T	T/T	C/T	ND	A	G		G	T	*4/*17
Case 11	C/T	G/A	C/T	T/T	C/T	ND	A	G		G	T	*4/*17
Case 12	C/T	G/A	C/T	T/T	C/T	ND	A	G		G	T	*4/*17
Case 13	C/T	G/A	C/T	T/T	C/T	ND	A	G		G	T	*4/*17
Case 14	C/T	G/A	ND	T/T	C/T	ND			ND			*4/*17
Case 15	C/T	G/A	ND	T/T	C/C	ND			ND			*4/*17
Case 16	C/T	G/A	ND	T/T	C/T	ND			ND			*4/*17
Case 17	C/T	G/A	ND	T/T	C/T	ND			ND			*4/*17
Case 18	C/T	G/A	ND	T/T	C/T	ND			ND			*4/*17
Case 19	C/T	G/A	C/T	C/T	C/T	ND	C	A		C	T	*4(D)/*17
Case 20	C/T	G/A	ND	C/T	C/T	ND			ND			*4(D)/*17
Case 21	C/T	G/A	C/T	C/T	C/T	C/T	C	A		C	T	*4Dx2/*17
Case 22	C/T	G/A	C/T	C/T	C/T	C/T	C	A		C	T	*4Dx2/*17
Case 23	C/T	G/A	C/T	C/T	C/T	ND	C	A		C	T	*4Dx2/*17
Case 24	C/T	G/A	ND	C/T	C/T	ND			ND			*4(D)/*17 [xN]
Case 25	C/T	G/A	ND	T/T	C/T	ND			ND			*4/*17 [xN]

ND, not determined. Sequencing results were obtained on a CYP2D6*4-specific template for cases 1–9 and 21–23; and deduced from diploid templates for cases 9, 10 and 19. *4(D) indicates that this subvariant is tentatively present. Alleles indicated as *4 were not further discriminated into *4A, B, F, G, H, J or *M*. *[xN]* indicates the presence of a duplication or multiplication event that was not further specified.

**Table 3:** Long-range PCR, TaqMan Assay and Sequencing details and conditions

Long-Range (XL)-PCR					
Fragment	Forward Primer (5′ to 3′)	Reverse Primer (5′ to 3′)	Annealing (°C)	Extension (min)	Fragment Length (kb)
A	ATGGCAGCTGCCATACAATCCACCTG	CGACTGAGCCCTGGGAGGTAGGTAG	68	7	6.6
D	CCAGAAGGCTTTGCAGGCTTCAG	CGGCAGTGGTCAGCTAATGAC	68	13	8.6 or 10.2
*4 allele specific	TGTGTGTGAGAGAGAATGTGTGCC	ACTGAGCCCTGGGAGGTAGGTAG	70	7.5	5.5

TaqMan Assays			
Variant	Position (M33388)	rs #	Assay ID
*2	2850	16947	C__27102425_10
*4	1846	3892097	C__27102431_D0
*10	100	1065852	C__11484460_40
*17	1023	28371706	C__2222771_40 (original)
*17	1023	28371706	C__2222771_A0 (alternative)

*CYP2D6* Gene Sequencing Primer (5′ to 3′)		
CMH	CAMH	TFS
CGAAATCGAGGATGAAGG	ACCTGGTCGAAGCAGTATGG	ACTCGCTGGCCTGTTTCA
GTCGTCCAAGGTTCAAATAGG	ACTCGCTGGCCTGTTTCAT	CCGGTGGTCGTGCTCAAT
CGCTGGCCTGTTTCATGTCCACG	GGGTCGTCCAAGGTTCAAAT	
CAATGGGCTTGAATTCCCACTGCCAAG		

## Notes

**Note:** The revised/alternative *CYP2D6*17* assay C___222771_A0 is now commercially available from Life Technologies Corporation at www.lifetechnologies.com.
